# Comparative Metagenome-Assembled Genome Analysis of “*Candidatus* Lachnocurva vaginae”, Formerly Known as Bacterial Vaginosis-Associated Bacterium−1 (BVAB1)

**DOI:** 10.3389/fcimb.2020.00117

**Published:** 2020-03-31

**Authors:** Johanna B. Holm, Michael T. France, Bing Ma, Elias McComb, Courtney K. Robinson, Aditya Mehta, Luke J. Tallon, Rebecca M. Brotman, Jacques Ravel

**Affiliations:** ^1^Institute for Genome Sciences, University of Maryland School of Medicine, Baltimore, MD, United States; ^2^Department of Microbiology and Immunology, University of Maryland School of Medicine, Baltimore, MD, United States; ^3^Department of Epidemiology and Public Health, University of Maryland School of Medicine, Baltimore, MD, United States

**Keywords:** women's health, gynecology, microbial genomics, odor, vaginal microbiome

## Abstract

Bacterial vaginosis-associated bacterium 1 (BVAB1) is an as-yet uncultured bacterial species found in the human vagina that belongs to the family *Lachnospiraceae* within the order *Clostridiales*. As its name suggests, this bacterium is often associated with bacterial vaginosis (BV), a common vaginal disorder that has been shown to increase a woman's risk for HIV, *Chlamydia trachomatis*, and *Neisseria gonorrhoeae* infections as well as preterm birth. BVAB1 has been further associated with the persistence of BV following metronidazole treatment, increased vaginal inflammation, and adverse obstetrics outcomes. There is no available complete genome sequence of BVAB1, which has made it difficult to mechanistically understand its role in disease. We present here a circularized metagenome-assembled genome (cMAG) of BVAB1 as well as a comparative analysis including an additional six metagenome-assembled genomes (MAGs) of this species. These sequences were derived from cervicovaginal samples of seven separate women. The cMAG was obtained from a metagenome sequenced with long-read technology on a PacBio Sequel II instrument while the others were derived from metagenomes sequenced on the Illumina HiSeq platform. The cMAG is 1.649 Mb in size and encodes 1,578 genes. We propose to rename BVAB1 to “*Candidatus* Lachnocurva vaginae” based on phylogenetic analyses, and provide genomic and metabolomic evidence that this candidate species may metabolize D-lactate, produce trimethylamine (one of the chemicals responsible for BV-associated odor), and be motile. The cMAG and the six MAGs are valuable resources that will further contribute to our understanding of the heterogeneous etiology of bacterial vaginosis.

## Introduction

Bacterial vaginosis (BV) is a common vaginal infection affecting approximately 30% of US reproductive-aged women, with both African- and Mexican-Americans disproportionately afflicted (Allsworth and Peipert, 2007; Koumans et al., 2007). Though treatable with antibiotics, BV has a high rate of recurrence (Bradshaw et al., 2006; Schwebke and Desmond, 2007). The microbiological diagnosis of BV is established using Nugent scoring of Gram-stained vaginal smears and is defined by a low abundance of *Lactobacillus* spp. morphotypes and a wide array of strict and facultative Gram-negative anaerobes (Nugent et al., 1991). The clinical diagnosis of BV is made when 3 of the 4 Amsel's criteria are met: vaginal pH > 4.5, homogenous vaginal discharge, a positive whiff test, and the presence of clue cells upon wet mount examination (Amsel et al., 1983). Aside from the burdensome symptoms of vaginal discharge and fishy odor, BV is also associated with increased risk to adverse health outcomes including preterm birth (Leitich et al., 2003), increased risk of sexually transmitted infections acquisition and transmission, including HIV (Taha et al., 1998; Cherpes et al., 2003; Ness et al., 2005; Atashili et al., 2008; Cohen et al., 2012) and pelvic inflammatory disease (Ness et al., 2004).

A critical step along the path to understanding the ecology and pathogenic potential of a bacterial species is the characterization of its genome. Yet many of the bacteria associated with BV have thus far been uncultivable, further complicating genome sequencing efforts. BV-associated bacterium 1 (BVAB1) is one such organism for which there is limited genetic information available. BVAB1 was first identified by Fredricks et al. (2005) using 16S rRNA gene amplicons Sanger sequencing of samples associated with BV, and has eluded cultivation efforts since. Based on the sequence of its 16S rRNA gene, BVAB1 belongs to the family *Lachnospiraceae* (Muzny et al., 2014) and has often been misidentified as belonging to the genus *Shuttleworthia* (Lamont et al., 2011; Petrova et al., 2013). Interestingly, Gram-negative curved rods designated *Mobiluncus* morphotypes on Gram stain in Nugent scoring have been shown to likely be BVAB1 (Srinivasan et al., 2013). Further, vaginal communities in which BVAB1 16S rRNA gene sequence is detected have been associated with vaginal inflammation and persistent BV in African women (Lennard et al., 2018). BVAB1 remains uncultured and aside from detection of this species via partial 16S rRNA gene amplicon sequencing, little is known about its metabolism, pathogenic potential, or ecology in the vaginal environment, especially during BV. Further understanding of the genetic and physiological properties of BVAB1 will help to dissect the complex etiology of BV. Previously, a 94 contig BVAB1 metagenome-assembled genome from short-read sequencing was produced (Fettweis et al., 2019).

In this study, we characterize the first circularized metagenome assembled genome (cMAG) of BVAB1 constructed from a metagenome sequenced using the PacBio Sequel II long read platform. In addition, we compare this cMAG to an additional six metagenome-assembled genomes (MAGs). All genomes originate from different women with symptomatic or asymptomatic BV. Based on phylogenetic analysis of full-length 16S rRNA gene sequences obtained from the genomic assemblies, we propose to rename this bacterium “*Candidatus* Lachnocurva vaginae”.

## Materials and Methods

### Sample Collection

Vaginal samples used in this study were identified as containing a high relative abundance (> 60%) of BVAB1 using 16S rRNA gene amplicon sequencing of the V3–V4 regions as previously reported (Holm et al., 2019). Cervicovaginal lavages from six participants that were collected as part of the NIH Longitudinal Study for Vaginal Flora (LSVF) (Klebanoff et al., 2004) by washing the vaginal walls with 3 mL sterile, deionized water, aspiration from the vaginal vault via pipette and stored at −80°C, were included in this study. Gram stain smears were prepared for Nugent scoring as previously described (Nugent et al., 1991). DNA was extracted from 200 μL of lavage fluid using the MagAttract Microbial DNA Kit (QIAGEN Inc., Germantown MD) automated on a Hamilton Star robotic platform according the manufacturer recommendations. DNA was eluted in a final volume of 110 μL nuclease-free water.

An additional swab sample collected as part of the UMB-HMP study was used in this study (Ravel et al., 2013). The swab was self-collected by a participant using a Copan Eswab re-suspended into 1 mL Amies transport medium (ESwab, Copan Diagnostics Inc.), frozen at −20°C for no more than a week, and then transferred to −80°C until analyzed. High molecular weight DNA was extracted from this sample using the MasterPure DNA purification kit (Lucigen) with two phenol/chloroform cleanups prior to DNA precipitation. DNA extraction was quantified on a TapeStation 2200 instrument run with a Genomic DNA tape (Agilent).

### Metagenomic Library Construction and Sequencing on the PacBio Sequel II Platform

The extracted DNA from the swab collected as part of the UMB-HMP study (Ravel et al., 2013) was found to be of sufficient concentration (5.49 ng/μL in 200 μL) for long-read sequencing using the Pacific Biosciences Sequel II platform (Pacific Biosciences). The sequencing library was prepared with SMRTBell Template Prep Kit 1.0 and was size-selected on a BluePippen (Sage Science) with a cutoff of 5 kb. The library was barcoded and sequenced as part of a multiplexed run with four other unrelated samples. Sequencing was performed on a PacBio Sequel II instrument with an 8M cell loaded at 60 pM at the Genomic Resource Center of the University Maryland School of Medicine.

### Long Read Quality Filtering, Host-Read Removal, and cMAG Construction

Raw reads were demultiplexed with *lima* (version 1.9.0) using default parameters except for minimum barcode score set at 26 and a minimum read length of 50 bp after clipping of the barcode was enforced. Both tools are part of the SMRTLink 6.0.1 software package with updated CCS version 3.4.1. Human reads were detected using pbalign v0.4.1 (Pacific Biosciences) and the human genome build 38 (GRCh38.p12). Remaining reads were corrected and assembled via Canu v1.7 and the “-pacbio-raw” protocol (Koren et al., 2017). The largest resulting contig was ca. 1.6 Mb in length, much larger than the second longest contig (ca. 600 kb). Four copies of the 16S rRNA gene were detected on this contig and were identical to the existing BVAB1 16S rRNA gene reference AY724739 Fredricks et al., 2005 (NEJM). The contig had 6.5 kb of overlapping ends and was determined to be circular by Canu. This contig was then manually circularized using Geneious version 2019.2.1 (Galens et al., 2011), searched for the *dnaA* gene, and rotated so that *dnaA* was the first gene (Kearse et al., 2012). The circularized metagenome-assembled genome (cMAG) was annotated using the IGS Prokaryotic Annotation Pipeline (Galens et al., 2011). Translated gene sequences were assessed against KEGG using the BlastKOALA algorithm for insights into “*Candidatus* Lachnocurva vaginae” metabolism (Kanehisa et al., 2016). Bacteriophages were detected using PHASTER (Zhou et al., 2011; Arndt et al., 2016). Completeness and contamination of the cMAG was analyzed using CheckM version 1.0.18 (Parks et al., 2015) and the taxonomy_wf flag specifying Order *Clostridiales* and rerun specifying Family *Lachnospiraceae*. Metabolic reconstruction was examined with the cMAG using the metabolic modeling function in KBase (Arkin et al., 2018). Complete media for gapfilling and a Gram-positive template were used.

### Metagenomic Library Construction and Sequencing on the Illumina HiSeq 4000 Platform

Metagenomic libraries for the six samples from the LSVF study were prepared using the KAPA HyperPlus Kit (Kapa Biosystems) with KAPA Single-Indexed Adapter Kit Set B. A fixed volume (35 μL) of genomic DNA was used as input, and libraries were prepared following the manufacturer's protocol with modifications based on their amount of input DNA as in Supplemental Data Sheet 1. For samples with 0.5 or 0.2 ng input DNA, the fragmentation enzyme was diluted 1:2 or 1:5 with water. All samples were fragmented at 37°C for 5 min. Adapter concentrations varied according to the input DNA as listed in Supplemental Data Sheet 1, and the adapter ligation was carried out overnight at 4°C for all samples. The post-ligation cleanup was performed with 0.8x Ampure XP beads (Beckman Coulter, Indianapolis IN) and 20 μL of sample was used in library amplification. Amplification library cycles varied by input DNA as listed in Supplemental Data Sheet 1. Post-amplification cleanup was performed with 1x Ampure XP beads; libraries with remaining adapter dimer peaks were cleaned a second time. The final elution was in 25 μL of nuclease-free water. Libraries were run on a TapeStation instrument with a D1000 tape (Agilent) to assess quality and concentration. Libraries were sequenced (8 libraries/lane) on an Illumina HiSeq 4000 instrument using the 150 bp paired-end protocol.

### Short Read Quality Filtering, Host-Read Removal, and Metagenomic-Assembled Genome Reconstruction

Metagenomic reads were quality filtered using Trimmomatic v0.36 (Bolger et al., 2014) to remove sequencing adapters allowing for 2 mismatches, a palindromic clip threshold of 30, and a simple clip threshold of 10. Bases with quality scores < 3 were removed from the beginning and end of reads combined with a 4 bp sliding window which trimmed a read if the average quality score within that window fell below 15. Reads < 75 bp in length were removed. Human reads were detected by mapping to the human genome build 38 (GRCh38.p12) with Bowtie 2 v2.3.4.1 and default settings (Langmead and Salzberg, 2012), and removed using samtools v1.9 (Li et al., 2009) and bedtools v2.27.1 (Quinlan and Hall, 2010; Quinlan, 2014) (see Supplemental Data Sheet 2 for specific scripts). Metagenomic assemblies were produced using SPAdes genome assembler v3.13.0 (Bankevich et al., 2012) with the careful setting. Resulting contigs were aligned to the cMAG using NUCmer with the –mum setting and allowing for 5,000 bp breaklength, filtered to remove aligned contigs < 100 bp, and minimum contig coverage of 25%. MAGs were annotated with the Live Annotate & Predict tool from Geneious version 2019.2.1 (Kearse et al., 2012) using the “*Ca*. Lachnocurva vaginae” cMAG as annotation source (sequence similarity of ≥ 95% required). Genes with no similarity to the cMAG were annotated with Prokka v1.13 (Seemann, 2014). Phages were detected using PHASTER (Zhou et al., 2011). A circle plot was constructed with the BLAST Ring Image Generator v0.95 (Alikhan et al., 2011), and the annotated cMAG.

### Metabolomic Analysis

Metabolomics analysis was conducted as previously described (Nelson et al., 2018) by Metabolon Inc. using 200 μL of each LSVF lavage sample used in this study or 200 μL of a frozen dry swab eluted in 1 ml of PBS for the sample from the UMB-HMP study (Ravel et al., 2013). The abundances of 561 compounds were quantified using Ultrahigh Performance Liquid Chromatography-Tandem Mass Spectroscopy (UPLC-MS/MS). Quantities were corrected for instrument block variability and reported as normalized area-under-the-curve estimates. Figures were generated using ggplot2 v3.2.0 (Wickham, 2009).

### Comparative Genome and Phylogenetic Analyses

Average nucleotide identities (ANI) were calculated using FastANI v1.1 with fragment lengths of 1,000 bp, which is about the mean length of coding sequences (Jain et al., 2018). Maximum-likelihood phylogenetic trees of full-length 16S rRNA gene sequences alignments were generated with MUSCLE v3.8.425 using default parameters (Edgar, 2004) and FastTree v2.1.11 using the Generalized Time Reversible model and default parameters (Price et al., 2009, 2010) in Geneious v11.0.3 (Kearse et al., 2012). The tree was rooted with *Fusobacterium nucleatum* (AJ133496) and *Propionigenium modestum* (X54275) (Domingo et al., 2009), and members of the genus *Lachnoclostridium* were included as neighbors (Yutin and Galperin, 2013) as well as 16S rRNA genes from *Shuttleworthia satelles* (NR_028827), and *Lachnospiraceae* bacterium 2_1_46FAA (NR_025127). In addition, the cMAG was submitted for taxonomic placement using the TrueBacID tool from EZBioCloud (Yoon et al., 2017). cMAG and MAG pseudomolecules were aligned and visualized using AliTV v1.0.6 (Ankenbrand et al., 2017). Genes were ordered by gene synteny of the cMAG. To examine homology to other reference sequences, the cMAG was aligned to the NCBI protein reference database (downloaded 1/28/2019), as well as the genomes of *Shuttleworthia satelles* (NZ_ACIP00000000), *Lachnobacterium bovis* (GCF_900107245.1), and *Lachnospiraceae* bacterium 2_1_46FAA (ADLB02000001.1) using BLAST v2.8.1+. The top 3 hits were chosen by lowest e-value, highest percent identity, and longest alignment length, in that order. Genomic and pathogenicity islands were explored using IslandViewer4 (Bertelli et al., 2017).

## Results and Discussion

### Participant Information

All six participants in the LSVF study were of reproductive age and had high Nugent scores (6–10) (Table 1). Four were diagnosed with asymptomatic Amsel-BV, one with symptomatic Amsel-BV, and one was negative for Amsel-BV (Mckinnon et al., 2019). The woman who participated in the UMB-HMP study (Ravel et al., 2013) had a Nugent score of 8 and was diagnosed with asymptomatic Nugent-BV. The vaginal microbiota of these 7 samples had >60% BVAB1 as defined by 16S rRNA gene V3-V4 amplicon sequencing (Ravel et al., 2013).

**Table 1:** Participants demographics and cervicovaginal lavage microbial compositions for samples used in metagenomic reconstruction of the “*Ca*. Lachnocurva vaginae” circularized metagenome-assembled genome using long-read (*) and shotgun sequencing (*).

**Sample ID**	**Age**	**Race**	**Microbiota Relative Abundances of Vaginal Samples (16S rRNA Gene –V3V4)**	**BV Status**	**Nugent Score**	**Vaginal pH**	**Clinician-Observed Discharge**	**Positive Whiff Test**
UAB071*	22	Black	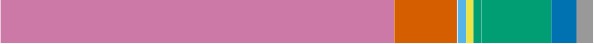	Asym	8	5	-	Yes
Y3207	37	Black	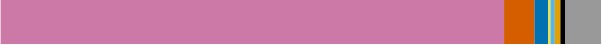	No	6	6.5	No	No
Y2266	34	Black	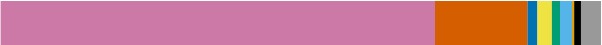	Asym	10	5.3	Mild visible—Gray/White	Yes
Y2337	36	Black	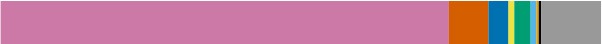	Asym	8	5.5	Mild visible—Gray/White	Yes
Y3255	24	Black	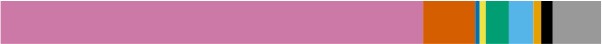	Asym	9	5.5	No	Yes
Y2624	27	Black	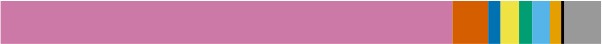	Asym	7	5.5	Mild visible—“Mucousy”	Yes
Y2694	34	White	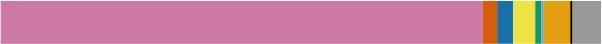	Sym	7	5.5	Mild visible—Gray/White	Yes

*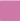, Ca.Lachnocurvavaginae; 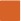, Gardnerellavaginalis; 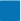, Megasphaera; 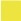, Sneathiasanguinegens; 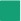, Prevotella; 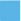, Atopobiumvaginae; 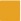, Prevotellaamnii; 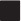, Mageeibacillusindolicus; 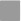, Other*.

*Other (gray, includes all taxa except the 8 most abundant taxa). Asym, asymptomatic BV; Sym, symptomatic BV*.

### Phylogenetic Analysis

**Figure 1:**
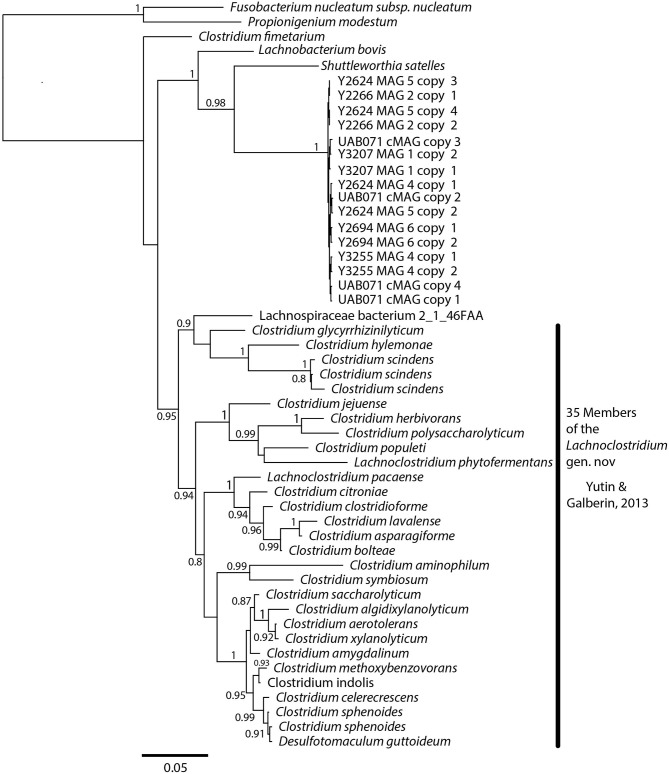
“*Ca*. Lachnocurva vaginae” is related to *Shuttleworthia satelles*; the full-length 16S rRNA gene sequences are 89–90% identical.

A phylogenetic analysis using the full-length 16S rRNA genes extracted from all MAGs revealed BVAB1 belongs in the *Clostridales* family *Lachnospiraceae*, and that *S. satelles* is the closest known relative (Figure 1), though nucleotide identity is only 89.2%. Similar results were obtained from EZBioCloud's TrueBacID (Supplemental Data Sheet 3). We therefore propose a new candidate species for BVAB1: “*Candidatus* Lachnocurva vaginae”, which represents the phylogenetic placement, the curved morphology of the cells, and the source of this cMAG. Considering the entire cMAG, the ANI between “*Ca*. Lachnocurva vaginae” and the *S. satelles* draft genome (NZ_ACIP00000000) was 81.85%. Relative to each other, “*Ca*. Lachnocurva vaginae” 16S rRNA genes were >99.7% identical and the cMAG and MAG average nucleotide identities were also high (98.6–99.2%, Table 2).

**Table 2:** Average Nucleotide Identity (ANI) between the “*Ca*. Lachnocurva vaginae” circularized metagenome-assembled genome, each metagenome-assembled genome, and the *Shuttleworthia satelles* draft genome NZ_ACIP00000000.

		**“*****Ca***. **Lachnocurva vaginae” MAG ID**							
		**Y2694_MAG_6**	**Y2624_MAG_5**	**Y3255_MAG_4**	**Y2337_MAG_3**	**Y2266_MAG_2**	**Y3207_MAG_1**	**UAB071**	
“*Ca*. Lachnocurva vaginae” MAG ID	Y2624_MAG_5	99.2	–	–	–	–	–	–	
		Y3255_MAG_4	99.0	99.01	–	–	–	–	–
		Y2337_MAG_3	98.95	98.99	98.97	–	–	–	–
		Y2266_MAG_2	99.06	99.01	98.93	99.05	–	–	–
		Y3207_MAG_1	98.98	98.95	99.07	98.99	99.06	–	–
		UAB071	99.15	99.00	98.92	99.09	99.13	98.98	–
		*Shuttleworthia satelles*	80.80	81.90	81.51	81.63	81.30	81.86	81.85

### Overall Genomic Features

**Figure 2:**
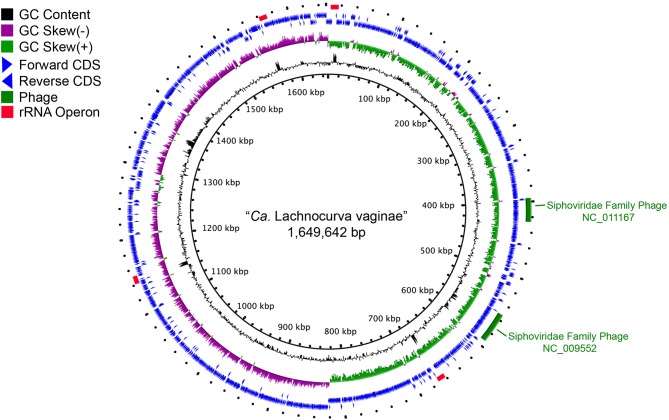
Circle plot of the “*Ca*. Lachnocurva vaginae” circularized metagenome-assembled genome (cMAG). Circles are from inside to outside: Circle 1: cMAG position; Circle 2: GC content, 3: GC skew, 4: forward-direction coding sequences, 5: reverse-direction coding sequences, and 6: phage detected in cMAG (green bars) and rRNA operons (red bars).

The cMAG of “*Ca*. Lachnocurva vaginae” is 1,649,642 bp in size with 31.8% GC content, encodes 1,578 genes and was estimated to be 99.1% complete and 0% contaminated at the order level and 90.9% complete and 0% contaminated at the family level. The relatively low completion estimate at the family level was due to 22 missing marker genes out of 312 (Supplemental Table 2). Mean coverage of the cMAG assembly by long reads was 124X (range: [26–285]). The “*Ca*. Lachnocurva vaginae” MAGs constructed using the short-read Illumina HiSeq 4000 metagenomic sequence data were 1.48–1.62 Mb in size (mean: 1.57 Mb) with a number of contigs ranging from 26 to 152 (mean N50: 99,943 bp) and 31.6% GC (Table 3). Genome annotation indicated the presence of four complete rRNA operon copies (Figure 2) and 42 tRNA genes in the cMAG. As expected, partial rRNA operons were identified in the Illumina-based MAGs (Supplemental Image 1).

**Table 3:** “*Ca*. Lachnocurva vaginae” MAG assembly characteristics.

**“*Ca*. Lachnocurva vaginae” MAG ID**	**No. CDS**	**Total Length (Mb)**	**N50**	**%GC**	**No. Contig**	**No. tRNAs**	**No. 5S rRNA**	**No. 16S rRNA**	**No. 23S rRNA**
UAB071	1,578	1.64	–	31.8	1	42	4	4	4
Y3207_MAG_1	1,463	1.60	42,408	31.6	152	41	1	1	1
Y2266_MAG_2	1,322	1.48	297,822	31.3	26	22	1	1	0
Y2337_MAG_3	1,492	1.62	60,576	31.6	143	42	0	0	0
Y3255_MAG_4	1,433	1.59	80,762	31.5	49	38	1	1	0
Y2624_MAG_5	1,459	1.61	168,048	31.8	31	43	2	2	2
Y2694_MAG_6	1,379	1.52	156,872	31.5	30	28	1	1	0

### Genomic Features of “*Ca*. Lachnocurva vaginae”

**Figure 3:**
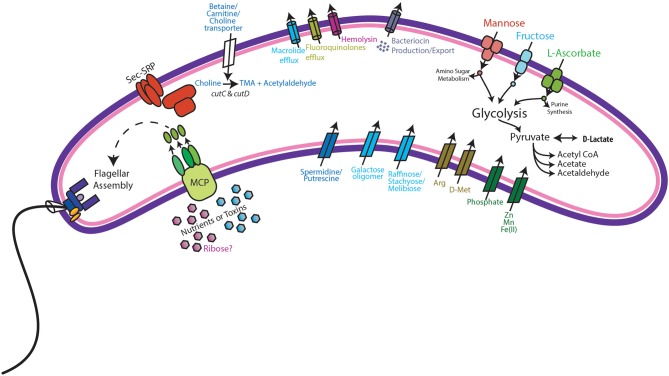
Genomic features of “*Ca*. Lachnocurva vaginae”. Genes coding for the following functions were observed: Methyl-accepting chemotaxis (MCP) and subsequent flagella assembly, Sec-SRP secretion systems, choline import, and metabolism to trimethylamine (TMA), bacteriocin production and export, mannose, fructose, and L-ascorbate transport and metabolism, and D-lactate dehydrogenase.

**Figure 4:**
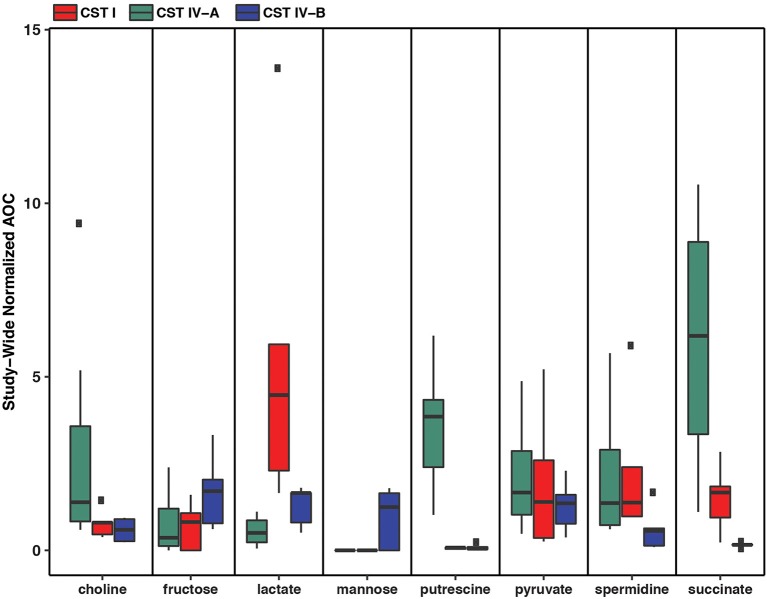
Metabolomic signatures for biochemicals of interest in vaginal community state type (CST) IV-A (high abundance of “*Ca*. Lachnocurva vaginae”), CST I (dominated by *Lactobacillus crispatus*), and CST IV-B (high abundance of *Gardnerella vaginalis*).

Metabolic modeling and reconstruction of the “*Ca*. Lachnocurva vaginae” cMAG contained 122 genes, 480 reactions, 581 metabolites, and no mass imbalance was found. Of the 1,578 genes detected, 255 genes had best blast matches of 80% sequence identity covering > 80% of the query gene. Of these, 165 matches were within the order *Clostridiales*, and 90 were not (Supplemental Image 2). Genes encoding transporters for mannose, fructose, and L-ascorbate were identified in all MAGs (Figure 3 and Supplemental Table 1, LCVA_199-201, LCVA_1491-1494), as were complete pathways for glycolysis, pyruvate oxidation, and the non-oxidative phase of the pentose phosphate pathway. Mannose was noticeably absent, or below the limit of detection, in the metabolome of all the samples, but present in other BV-like samples that did not contain a high relative abundance of the candidate species (Figure 4). We found “*Ca*. Lachnocurva vaginae” to have the genetic capability to uptake and metabolize mannose (**LCVA_1184**), suggesting mannose could also be a carbohydrate source for the candidate species (Figure 3).

The D-lactate dehydrogenase gene (**LCVA_41**), but not an L-lactate dehydrogenase gene was also observed in all MAGs. This result was unexpected, as the production of D-lactate in the vaginal environment is thought to be a key and somewhat unique feature of certain *Lactobacillus* spp. (Witkin et al., 2013; France et al., 2016). Metabolomic data for each sample were explored for the presence of lactate to provide evidence supporting this genomic finding. While lactate was observed in most samples, its abundance was substantially lower than that from representative samples that were dominated by *Lactobacillus crispatus*, a known D-lactate producer (Figure 4). Thus, either the enzyme exhibits lower activity in “*Ca*. Lachnocurva vaginae” or is instead used in the reverse reaction to consume *Lactobacillus* spp.-produced D-lactate to produce pyruvate. This is a strategy described for another vaginal anaerobic Gram-negative coccus, *Veillonella parvula*, which is able to grow on lactate as the sole source of carbon (Gronow et al., 2010), and would possibly contribute to an increase in vaginal pH, thereby creating a more favorable growth environment. However, for lactate to convert to pyruvate in anaerobic bacteria, an electron-bifurcating complex with an electron transfer flavoprotein alpha and beta subunit (EtfAB) would be necessary to make the reaction favorable (Weghoff et al., 2015). An EtfAB homolog was not observed in the “*Ca*. Lachnocurva vaginae” cMAG, suggesting that another mechanism may be involved. Instead, succinate was the most abundant short chain fatty acid in the communities from which “*Ca*. Lachnocurva vaginae” MAGs originated (Figure 4). Not surprisingly, succinate has been associated with bacterial vaginosis, a condition in which “*Ca*. Lachnocurva vaginae” is often found (Srinivasan et al., 2015). Interestingly, all “*Ca*. Lachnocurva vaginae” MAGs lacked genes for all steps of the tricarboxylic acid cycle, indicating it is unable to produce succinate via this pathway. It is possible that “*Ca*. Lachnocurva vaginae” produces metabolite(s) other species in the community can convert into succinate or produces it via a yet to be determined pathway. While “*Ca*. Lachnocurva vaginae” does not seem to be able to produce succinate, it has the genetic machinery to produce acetate from pyruvate (i.e., *ackA* and *pta* **LCVA_939** and **LCVA_1006**). We were, however, unable to detect acetate in the metabolome through the methods used, as it is too small of a molecule.

**Figure 5:**
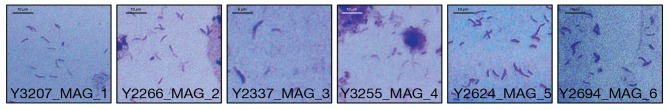
Gram stains of LSVF samples containing >70% “*Ca*. Lachnocurva vaginae” and used in this study. Morphologically “*Ca*. Lachnocurva vaginae” is a curved rod. Images are labeled with the sample ID.

A full suite of genes required for flagella assembly was observed in all MAGs, as well as genes required for methyl-accepting chemotaxis (**LCVA_205, LCVA_992, LCVA_1053, LCVA_1122)** and for the downstream signal process that mediates flagellar response, *cheY* (**LCVA_362**, **LCVA_688)** (Bren and Eisenbach, 2000). Flagella have yet to be visually observed on “*Ca*. Lachnocurva vaginae” and were not observed in the source samples used for this study (Figure 5).

We found the cMAG and some MAGs to also include genes that encode protection against antibiotics including the *tetO* gene (**LCVA_1310**, also observed in MAG Y3255) and drug efflux pumps for macrolides (*macB* **LCVA_500**, also observed in MAGs Y2337 and Y2266) and fluoroquinolones (**LCVA_1202**, present in all MAGs). A gene for hemolysin, *tlyA*, was also observed in all MAGs (**LCVA_1031**), suggesting direct interaction between “*Ca*. Lachnocurva vaginae” and host tissues.

Additionally, choline transporters, as well as the *cutC* and *cutD* genes were detected in all MAGs (**LCVA_1194** and **LCVA_1193**, respectively). The *cut* genes metabolize choline and produce trimethylamine (TMA) (Martinez-Del Campo et al., 2015). Aside from transport of exogenous choline, another potential source of choline would be via phospholipase D hydrolysis of phosphotidyl choline into choline as seen in gut bacteria (Chittim et al., 2019), however no genes encoding such activity were found in the cMAG of “*Ca*. Lachnocurva vaginae”. Comparing the translated *cutC* amino acid sequence from “*Ca*. Lachnocurva vaginae” to those reported by Martinez-Del Campo et al. (2015), we observed three of the five active sites conserved in “*Ca*. Lachnocurva vaginae” (Cys489, Glu491, Gly821, data not shown) indicating that it is likely functional. TMA is one of the substances believed to be responsible for the fishy odor associated with BV (Brand and Galask, 1986; Wolrath et al., 2002), however it is rarely detected in metabolomics analyses as it is highly volatile, unless it is performed on freshly collected samples (Wolrath et al., 2002).

Two intact bacteriophages were detected in the cMAG (Figure 2). Partial matches to the same phages were observed in all MAGs (Supplemental Image 1). Best BLAST hits indicated that both bacteriophages belong to the Siphoviridae family of double-stranded viruses (NC_009552, NC_011167) which can exhibit both lytic and lysogenic phases. Bacteriophages of this family have previously been reported in the vaginal species *Lactobacillus jensenii* (Martin et al., 2010). Genomic islands were detected at multiple sites (Supplemental Table 1 and Figure 2). Analysis of proteins encoded in the largest island (65.5 kb, coordinates 1,331,959–1,391,037, **LCVA_1280-1330**) showed similarity to proteins from *Shuttleworthia satelles*, and other taxa from the *Clostridiales* (Supplemental Table 3 and Supplemental Image 2) and several transposons and integrases, as well as tetracycline resistance proteins (TetO, **LCVA_1310**), and ABC transporters. We ruled out the presence of genomic islands due to mis-assembly by analyzing the long-read coverage spanning the 5′ and 3′ ends of genomic islands. More than 100 long PacBio reads of a means size of 9 kb span the junctions of all detected genomic islands. Further, mean coverage of the genomic islands was similar to that of the mean cMAG coverage of 124X. Portions of these islands were observed in the other MAGs assembled in this study (Supplemental Image 1 and Supplemental Table 1). The stringency of the mapping would not recover reads to regions of sequence diversity, thus these results may indicate this “*Ca*. Lachnocurva vaginae” likely contains multiple regions of genetic fluidity or diversity. However, the missing regions in MAGs may also be an artifact of metagenomic assembly.

## Conclusion

We present here a circularized MAG of “*Ca*. Lachnocurva vaginae” and six MAGs of the candidate species, previously known as BVAB1, an important member of the human vaginal microbiota associated with bacterial vaginosis and other adverse outcomes. Short-read metagenomic assembles do not perform well and lead to sub-optimal assemblies with missing regions, whereas long read metagenomic assemblies are promising and can generate circularized metagenome assembled genomes, as shown in this study. Our inability to culture this bacterium has limited our understanding of its ecological role in the vaginal environment and its relation to women's health. We have shown that “*Ca*. Lachnocurva vaginae” has the genomic potential for motility and chemotaxis, and is likely capable of resisting several antibiotics via drug efflux systems. Our analysis indicates this candidate species may contribute to the fishy odor characteristic of bacterial vaginosis through the production of TMA from choline. This crucial genomic data could be used in metabolic modeling experiments to define a culture medium suitable for the cultivation of “*Ca*. Lachnocurva vaginae”, a critical step to further understand its role in the vaginal microbiome.

## Data Availability Statement

Sequence data have been submitted to NCBI under BioProject PRJNA562728.

## Ethics Statement

Samples used in this study were archived and de-identified cervicovaginal lavages and swabs. The samples were originally collected after obtaining informed consent by all participants, who also provided consent for storage of the samples and use in future research studies related to women's health. The original study was approved by the University of Maryland School of Medicine Institutional Review Board.

## Author Contributions

EM and CR performed the extraction and library preparations from the raw samples. JH, AM, LT, MF, and BM performed bioinformatics analyses to generate the MAGs. JH, RB, and JR designed the study. JH, MF, BM, and JR executed the study, contributed to the analyses. JH, RB, MF, BM, and JR wrote and edited the manuscript.

### Conflict of Interest

JR is co-founder of LUCA Biologics, a biotechnology company focusing on translating microbiome research into live biotherapeutics drugs for women's health. The remaining authors declare that the research was conducted in the absence of any commercial or financial relationships that could be construed as a potential conflict of interest.

## Supplementary Material

Supplemental Data Sheet 1 Metagenomic library preparation modifications based on input amounts of DNA.

Supplemental Data Sheet 2 Processing scripts for analysis.

Supplemental Data Sheet 3 Taxonomic and phylogenetic placement of “Ca. Lachnocurva vaginae” (UAB071) from EZBioCloud TrueBacID system.

Supplemental Table 1 List of genes annotated in the “Ca. Lachnocurva vaginae” cMAG. Excludes rRNA and tRNA.

Supplemental Table 2 List of marker genes used in Family-level CheckM analysis and presence (1) or absence (0) in the “Ca. Lachnocurva vaginae” cMAG.

Supplemental Table 3 Top 3 best BLAST matches to the genes of the “Ca. Lachnocurva vaginae” cMAG.

Supplemental Image 1 Visualization of “Ca. Lachnocurva vaginae” whole genome alignments of cMAG and other MAGs.

Supplemental Image 2 Circle plot of “Ca. Lachnocurva vaginae” cMAG with best BLAST hits indicated as either Clostridiales or not (Ring 5). If hit was within Clostridiales and >80% identical to cMAG, the specific taxon of close relatives was indicated in Ring 4. Included BLAST hits covered at least 80% of the query gene.
