# Supplementary material for: Comparative Metagenome-Assembled Genome Analysis of “Candidatus Lachnocurva vaginae”, Formerly Known as Bacterial Vaginosis-Associated Bacterium−1 (BVAB1)
Source: Front Cell Infect Microbiol. 2020 Mar 31;10:117. doi: 10.3389/fcimb.2020.00117 (PMC7136613; doi:10.3389/fcimb.2020.00117)
Supplement: Supplemental Data Sheet 1 — Metagenomic library preparation modifications based on input amounts of DNA. [file Data_Sheet_1.docx]

Supplementary Data Sheet 1. Modifications to metagenomic library preparation as determined by input DNA concentrations.

| **Target input DNA (ng)** | **Range input DNA (ng)** | **Fragment enzyme dilution** | **Adapter concentration (**µM) | **Amplification cycles** |
| --- | --- | --- | --- | --- |
| 250 | 175-325 | - | 15 | 5 |
| 100 | 75-175 | - | 15 | 6 |
| 50 | 37.5-75 | - | 15 | 8 |
| 25 | 17.5-37.5 | - | 7.5 | 10 |
| 10 | 7.5-17.5 | - | 3 | 13 |
| 5 | 3.75-7.5 | - | 1.5 | 14 |
| 2.5 | 1.75-3.75 | - | 0.75 | 16 |
| 1 | .75-1.75 | - | 0.3 | 19 |
| 0.5 | .4-.75 | 1:2 | 0.3 | 19 |
| 0.2 | .15-.4 | 1:5 | 0.3 | 19 |
