# Supplementary material for: Comparative Metagenome-Assembled Genome Analysis of “Candidatus Lachnocurva vaginae”, Formerly Known as Bacterial Vaginosis-Associated Bacterium−1 (BVAB1)
Source: Front Cell Infect Microbiol. 2020 Mar 31;10:117. doi: 10.3389/fcimb.2020.00117 (PMC7136613; doi:10.3389/fcimb.2020.00117)
Supplement: Supplemental Data Sheet 2 — Processing scripts for analysis. [file Data_Sheet_2.docx]

Supplementary Data Sheet 2

## Quality filtering

java -jar /usr/local/packages/trimmomatic-0.36/trimmomatic-0.36.jar PE -phred33 -threads 4 $R1 $R2 -baseout $baseout ILLUMINACLIP:/usr/local/packages/trimmomatic/adapters/TruSeq3-PE-2.fa:2:30:10 LEADING:3 TRAILING:3 SLIDINGWINDOW:4:15 MINLEN:75

## Host-read detection

/usr/local/packages/bowtie2-2.3.2/bowtie2 -x /local/projects-t2/jholm/human_genome/GCA_000001405.15_GRCh38_no_alt_analysis_set.fna.bowtie_index -1 $P1 -2 $P2 -S $bowtieOut

## Host-read removal

samtools view -bS $bowtieOut -o $samOut1 > $samOut1

samtools view -b -f 4 -F 256 $samOut1 -o $samOut2

samtools sort -n $samOut2 -o $samOut3

bamToFastq -i $samOut3 -fq $hostRm -fq2 $hostRm2

## Above repeated for the unpaired reads resulting from trimmomatic -> "qhostRm_se"

## De-novo assembly with Spades and trusted-contigs setting

System information:

SPAdes version: 3.13.0

Python version: 2.7.14

OS: Linux-3.10.0-693.5.2.el7.x86_64-x86_64-with-redhat-7.4-Maipo

/usr/local/bin/spades.py -k 21,33,55,77 --careful --trusted-contigs $closed_genome -1 $hostRm -2 $hostRm2 -s $hostRm_se -o $spades_out

## Read mapping to assembly

bowtie2-build -f $closed_genome $closed_genome_bt

/usr/local/packages/bowtie2-2.3.2/bowtie2 -1 $hostRm -2 $hostRm2 -x $id_bt -S $id_mapping.sam

## Alignment of spades output (scaffolds.fasta -> $contigs) to closed genome, filtering of aligned contigs, and production of contig and pseudomolecule file.

nucmer -p nucmer --mum -b 3500 $closed_genome $contigs

delta-filter -qr -l 100 nucmer.delta > nucmer.filter.delta

show-coords -THrcl nucmer.filter.delta > nucmer.filter.coords

show-tiling nucmer.filter.delta > nucmer.filter.tiling

nucmer2psuedo.pl -i ../scaffolds.fasta -l nucmer.filter.tiling -o $MAG_contigs

### fastANI

for f in *_contigs.fasta; do fastANI --refList list_of_contig_files.txt -q $f -o ${f/_contigs.fasta}_ani;done

fastANI --refList list_of_ps.txt -q ../S_satelles_GCF000160115.fasta -o shuttleworthia_ani --fragLen 100

### cgview for BRIG

Figure 2: java -Xmx1500m -jar ~/Downloads/BRIG-0.95-dist/cgview/cgview.jar -f png -i /Users/johannaholm/BRIG_OUT/scratch/Figure_2.xml -o /Users/johannaholm/BRIG_OUT/scratch/Figure_2.png -H 3000 -W 3000

Supp. Fig. 1: java -Xmx1500m -jar ~/Downloads/BRIG-0.95-dist/cgview/cgview.jar -f svg -i /Users/johannaholm/23Jan2020/scratch/Supplemental_Figure_1.xml -o /Users/johannaholm/sf1.svg -H 3500 -W 3500

### CheckM

checkm taxonomy_wf family Lachnospiraceae . checkm_family_Lachnospiraceae -x .fasta -t 4

checkm taxonomy_wf order Clostridiales . checkm_order_Clostridiales -x fasta -t 4

checkm qa checkm_family_Lachnospiraceae/Lachnospiraceae.ms checkm_family_Lachnospiraceae -o 4 > checkm_family_Lachnospiraceae-out.txt

### blastp for cMAG translated gene sequences

blastp -query UAB071.faa -db /local/db/ncbi/blast/db/nr -outfmt \"6 qseqid sseqid staxid qlen slen qstart qend length evalue pident\"

### Mean gene coverage

bedtools coverage -a <bed file> -b <bam file> -mean > out.txt

## Nucmer + Mummerplot

nucmer UAB071.fasta PQVO01.1.fsa_nt

mummerplot out.delta.m -R UAB071.fasta -Q PQVO01.1.fsa_nt --filter --layout -p PQVO01_to_UAB071 --png

mummerplot out.delta -R UAB071.fasta -Q PQVO01.1.fsa_nt --filter --layout -p PQVO01_to_UAB071 --png -x [1300000:1400000]
