# Supplementary figures and images for: Comparative Metagenome-Assembled Genome Analysis of “Candidatus Lachnocurva vaginae”, Formerly Known as Bacterial Vaginosis-Associated Bacterium−1 (BVAB1)
Source: Front Cell Infect Microbiol. 2020 Mar 31;10:117. doi: 10.3389/fcimb.2020.00117 (PMC7136613; doi:10.3389/fcimb.2020.00117)

Genomic Island  
Phage  
rRNA Operon

"Ca. Lachnocurva vaginae"  
cMAG

Y3207\_MAG\_1

Y2266\_MAG\_2

Y2337\_MAG\_3

Y3255\_MAG\_4

Y2624\_MAG\_5

Y2694\_MAG\_6

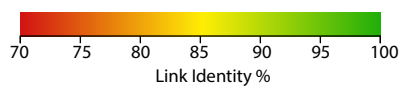

Supplement: Supplemental Image 1 — Visualization of “Ca. Lachnocurva vaginae” whole genome alignments of cMAG and other MAGs. [file Image_1.pdf]
