# Supplementary material for: Comparative Metagenome-Assembled Genome Analysis of “Candidatus Lachnocurva vaginae”, Formerly Known as Bacterial Vaginosis-Associated Bacterium−1 (BVAB1)
Source: Front Cell Infect Microbiol. 2020 Mar 31;10:117. doi: 10.3389/fcimb.2020.00117 (PMC7136613; doi:10.3389/fcimb.2020.00117)
Supplement: Supplemental Image 2 — Circle plot of “Ca. Lachnocurva vaginae” cMAG with best BLAST hits indicated as either Clostridiales or not (Ring 5). If hit was within Clostridiales and >80% identical to cMAG, the specific taxon of close relatives was indicated in Ring 4. Included BLAST hits covered at least 80% of the query gene. [file Image_2.pdf]

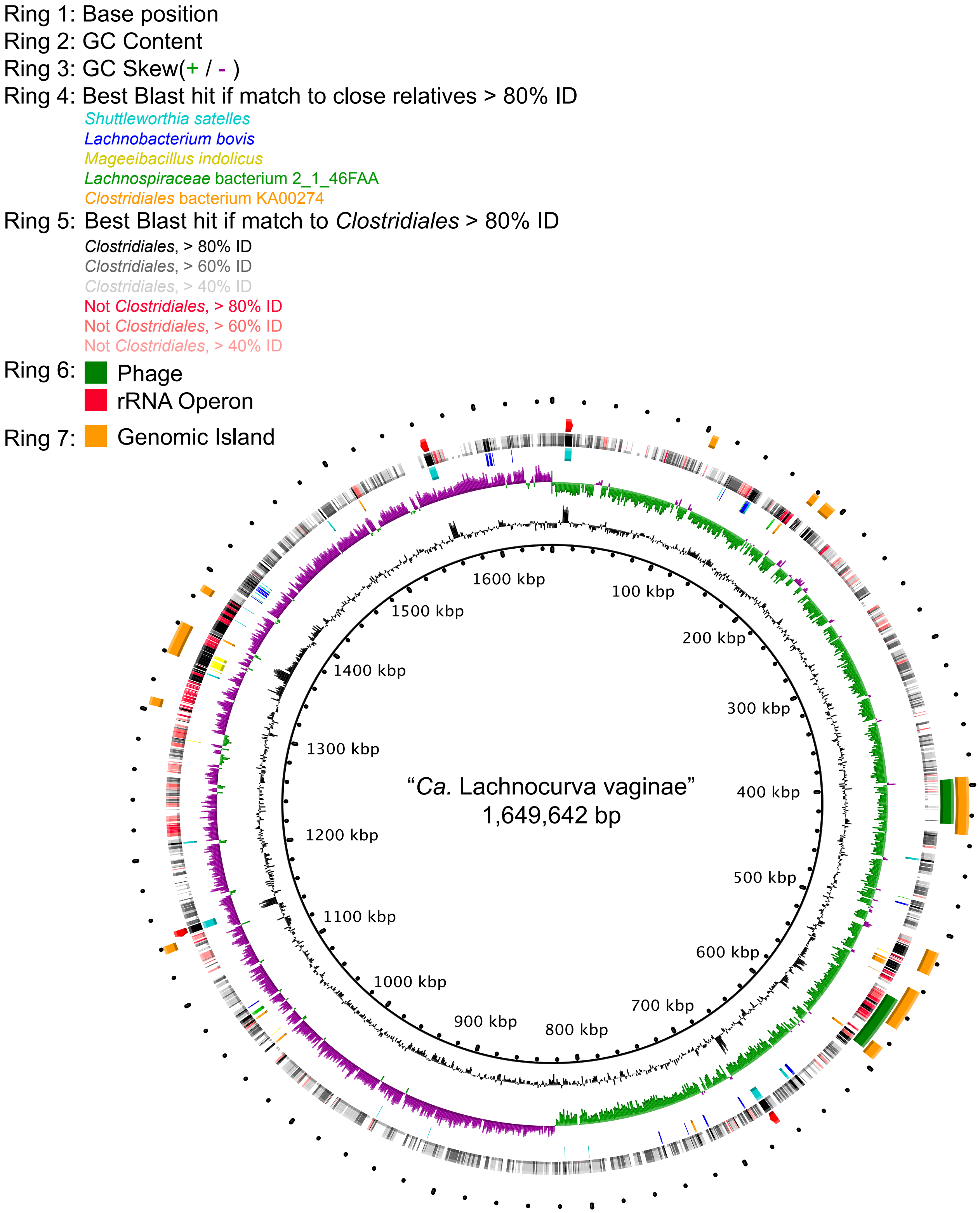

Supplemental Figure 2. Circle plot of "*Ca.* Lachnocurva vaginae" cMAG with best blast hits indicated as either *Clostridiales* or not (Ring 5). If hit is within *Clostridiales* and >80% ID, specific taxon of close relatives is indicated in Ring 4. Included BLAST hits covered at least 80% of the query gene.
